# Green sonochemistry assisted synthesis of hollow magnetic and photoluminescent MgFe_2_O_4_–carbon dot nanocomposite as a sensor for toxic Ni(ii), Cd(ii) and Hg(ii) ions and bacteria

**DOI:** 10.1039/d1ra02458b

**Published:** 2021-06-28

**Authors:** Shahla Ahmadian-Fard-Fini, Davood Ghanbari, Omid Amiri, Masoud Salavati-Niasari

**Affiliations:** Institute of Nano Science and Nano Technology, University of Kashan P. O. Box 87317-51167 Kashan Iran salavati@kashanu.ac.ir; Department of Science, Arak University of Technology Arak Iran d-ghanbari@arakut.ac.ir; Faculty of Chemistry, Razi University Kermanshah 6714414971 Iran; Department of Chemistry, College of Science, University of Raparin Rania Kurdistan Region Iraq

## Abstract

The purpose of this study was synthesis of photoluminescent nanoparticles for detection of toxic metal ions. Also, these controllable magnetic nanocomposites were used for detection of *Pseudomonas aeruginosa* bacteria. Carbon nano-templates were formed by calcination and sonication of lemon extract as a bio-compatible precursor. Then MgFe_2_O_4_ nanoparticles were incorporated on the carbon nano-templates. The composite was calcinated to decompose carbon and obtain hollow structures. Finally, photoluminescent carbon dots were deposited on the porous magnesium ferrite core. Because of the hollow structure, carbon dots can diffuse to the Mg-ferrite core so magnetic and photoluminescence properties are available simultaneously. Photoluminescence intensity decreases with increasing Ni(ii), Cd(ii), Hg(ii) metal ions and *Pseudomonas aeruginosa*. Results show an effective nanostructure for identification of toxic metal ions and also bacteria.

## Abstract 2

The purpose of this study is synthesis of magnetic and photoluminescence nanoparticles at the same time for finding of bacteria that could be fatal and its detection are crucial for some illness prohibition.

## Graphical abstract

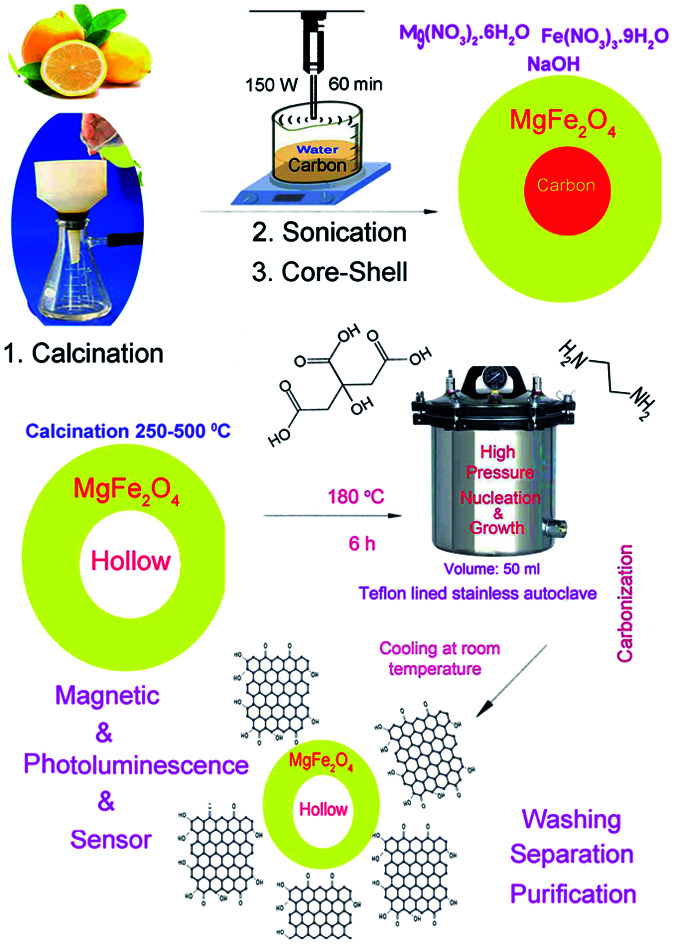

## 1. Introduction

Carbon dots (CDs) originating from graphene have both sp^3^ and sp^2^ hybridization (with carbon–oxygen or hydrogen–oxygen functional groups). Relative to typical quantum dots, CDs are desirable substitutes considering their chemical durability and lack of harmful components.^1,2^ The main properties of CDs are easy electron transportation, low price, controllable photoluminescence (PL), multi-photon stimulation, broad range light emission, easy functionalization, dispersibility in polar solvents, and stability against photo-bleaching and photo-blinking. Luminescence may originate from defects and traps, quantum-confinement of excitons, and also ring structures.^3–6^ Green fluorescence can be used as a sensor in cell imaging and other biomedical applications.^7–12^

Sono-chemistry at frequencies higher than 20 kHz has many applications in, for example, structural analysis, determination of distance, sono-photographic healing, removing dirt, and transfer of information. When sound waves with frequencies higher than 20 000 MHz e a liquid, they can break chemical bonds (sonolysis) and produce free radicals. In sono-chemistry, nucleation, cavitation, bubble interactions and effective chemical processes occur. Sono-chemistry is helpful in enhancing the rate of catalytic reactions, increasing degradation of pollutants (like pharmaceutical waste), and preparation of new materials.^13–15^ Ultrasonication leads to high pressure in liquids (compression); increase in pressure causes bubbles to become smaller, and lowering of pressure leads to expansion of bubbles. Bubbles become larger in a widespread way, with cycles until the ultrasound energy can no more be captured and the forces lead to strong breakdown. In water as solvent, reduction–oxidation reactions occur and hydrogen and hydroxyl radicals are produced.^16–18^

A lot of industrial and urban pollutants have been covertly added to pure water sources, one of the main group of toxic pollutants being heavy metal ions, which can have harmful effects on biological systems. As we know, these toxic ions can cause various mutations in biological systems, so portable and easy detection of them is necessary for protecting community health. Bio-compatible magnetic materials like magnetite have attracted much attention for use as a core for accommodating nanostructures; in this work another biocompatible material, magnesium ferrite, was selected based on its reported appropriate properties.^19,20^

Commonly, bacterial testing requires time and pricey instruments for identification, which is not acceptable in disease emergency conditions, so rapid recognition of bacteria is important for prevention of contagion. *Pseudomonas aeruginosa* is resistant to antibiotics; this bacterium can develop in low-oxygen atmospheres, it can cause disease in people, and it is known to appear in every part of the body, notably in healing wounds. If essential organs are colonized, infection can even be fatal.^21–23^ In this work, fluorescent hollow magnesium ferrite-CDs were prepared for rapid identification of bacteria, using sono-chemical and hydrothermal treatment.

## 2. Experimental

### 2.1. Synthesis of carbon dots

Ethylene diamine (0.6 g, 0.01 mol) and citric acid monohydrate (2.1 g, 0.01 mol) were dissolved in 500 ml of water. Next, the solution was autoclaved (Teflon-lined stainless steel) at 200 °C for 24 h. The final product was dispersed in water using ultra-sonic radiation (100–150 W, 30–60 min).

### 2.2. Synthesis of MgFe_2_O_4_ nanostructures

Carbon (as template for giving a particular shape) was obtained by calcination (350 °C, 2 h) of lemon extract. To eliminate agglomeration the product was dispersed in water under sonication (150 W, 1 h). Next, 0.2 g of the carbon, 0.02 mol of Mg(NO_3_)_2_·6H_2_O and 0.04 mol of iron nitrate·9H_2_O were added to water (200 ml). Sodium hydroxide solution (1 M, 15 ml) was added to adjust the pH to 11. After that the reaction mixture was autoclaved at 200 °C for 12 h; then the gray product was collected and washed with de-ionized water and ethanol. Finally, the compound was calcinated at 600 °C for 3 h for carbon decomposition. 0.2 g of magnesium ferrite was dispersed in 50 ml of water. Also, for suitable dispersion and to prevent agglomeration, ultra-sonic waves were applied (100–150 W, 30–60 min).

### 2.3. MgFe_2_O_4_–carbon dot nanostructures

Magnesium ferrite (0.2 g) was dispersed in 50 ml of de-ionized water and then both citric acid and ethylene diamine (0.02 mol) were dissolved and the solution was autoclaved (Teflon-lined stainless steel) at 200 °C for 24 h; it was then centrifuged and the solid product was washed with ethanol.

## 3. Results and discussion

**Fig. 1:**
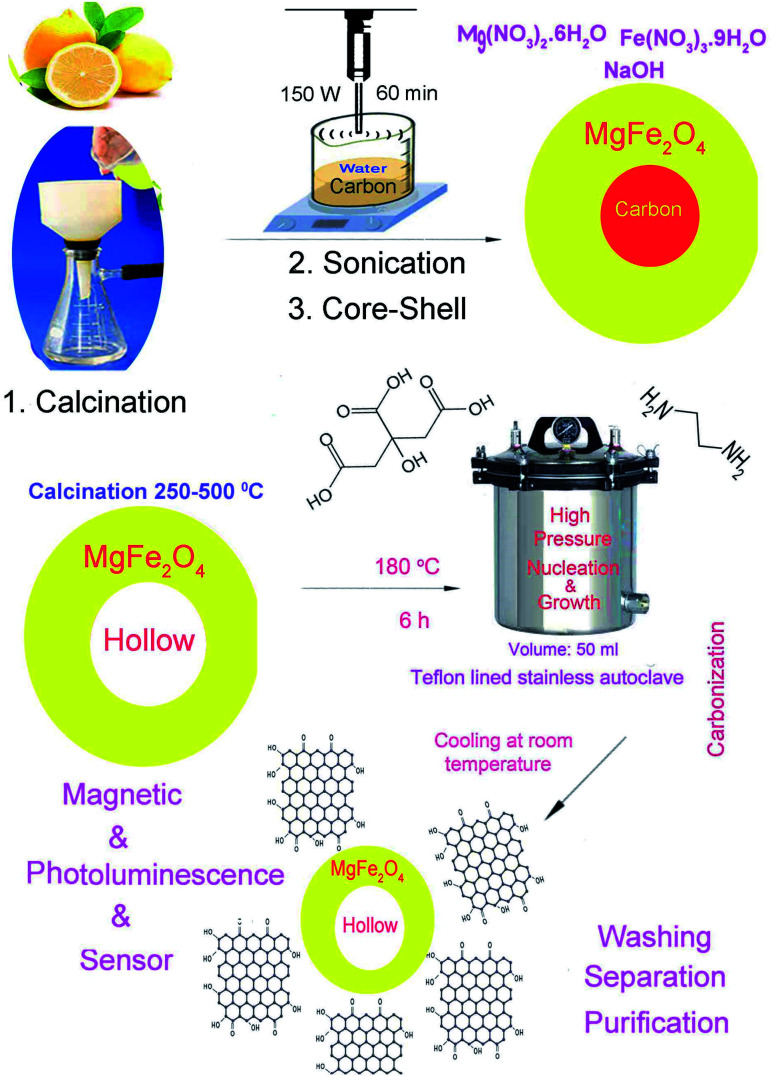
Schematic of preparation of carbon dots and hollow magnetic nanocomposite.

**Fig. 2:**
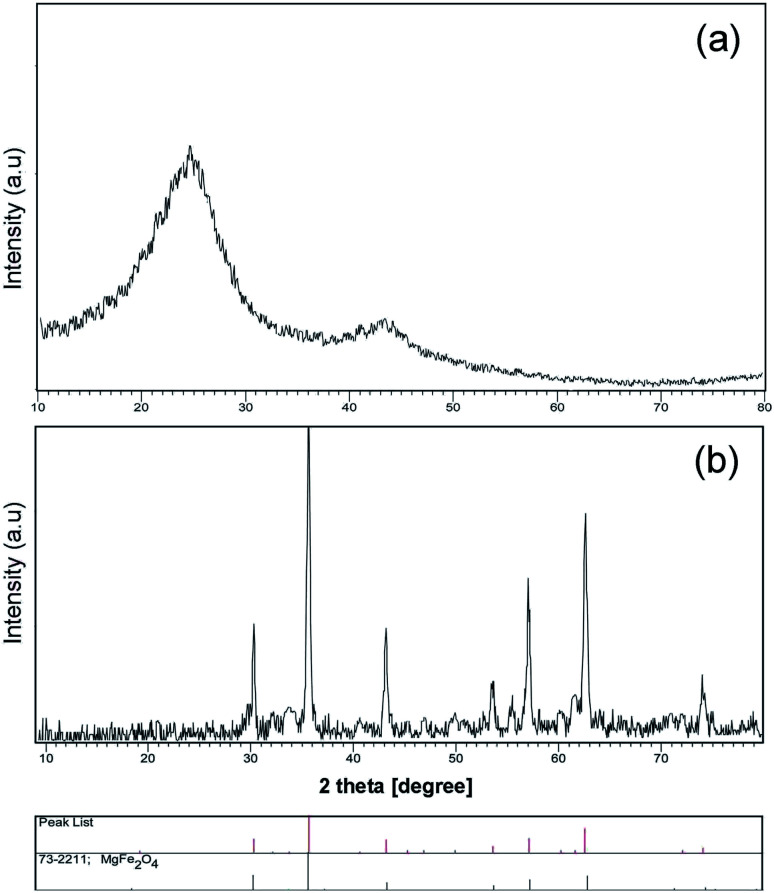
XRD patterns of (a) carbon dot nanoparticles, and (b) MgFe_2_O_4_–carbon dot nanocomposite.

A schematic of the synthesis of MgFe_2_O_4_–carbon dots by the solvo-thermal method is depicted in Fig. 1. Brunauer–Emmett–Teller (BET) results for the prepared carbon dots are shown in Table 1. Fig. 2a shows the X-ray diffraction spectrum of carbon dots obtained at 200 °C for 6 h, which is in agreement with the literature, and shows an amorphous semi-crystalline pattern.^1,3,4^Fig. 2b displays the X-ray diffraction (XRD) pattern of the magnesium ferrite–carbon dot product. The inverse spinel (phase: cubic) structure of MgFe_2_O_4_ (73-2211 JCPDS number, number 227 of *Fd*3̄*m* space group) was confirmed.

**Table 1:** BET results of carbon dots

BET plot		
*V*_m_	7.64	[cm_(STP)_^3^ g^−1^]
*a*_s,BET_	33.35	[m^2^ g^−1^]
*C*	151.07	
Total pore volume (*p*/*p*_0_ = 0.990)	0.944	[cm^3^ g^−1^]
Average pore diameter	9.39	[nm]

The luminescence of the CDs samples was investigated using ultra-violet irradiation. Samples are yellow under ambient light; however, they are green under UV exposure.

**Fig. 3:**
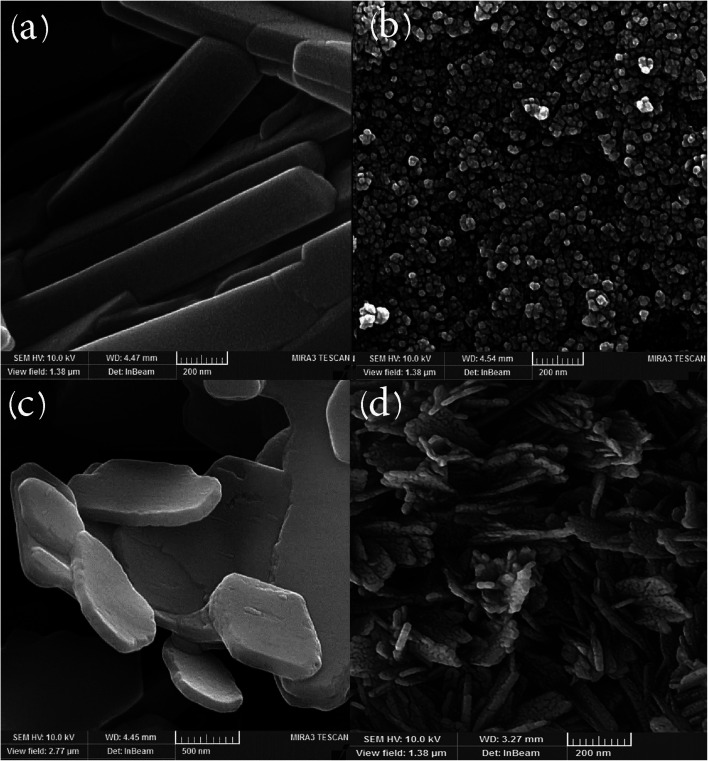
SEM images of (a) tetragonal carbon template prepared by calcination and (b) by sono-chemical method; (c) hexagonal MgFe_2_O_4_ prepared by hydrothermal method; (d) MgFe_2_O_4_ nanoparticles after calcination and sonication.

Fig. 3a displays a scanning electron microscopy (SEM) image of the carbon template obtained from lemon extract calcination (green precursor), which confirms preparation of tetragonal structures; according to the images obtained, the average diameter is about 200 nm. High temperature leads to agglomeration and aggregation, so in this work ultrasound radiation was also used to prepare mono-disperse nanoparticles. Three locations for reaction exist in sono-chemistry: the gas in the bubbles (pyrolysis), the surface of bubbles (reactions happen in *P*–*T* gradients) and the mass solution (energy is transferred to the solvent and multiplies the bubbles). Temperature, pressure, frequency, vessel shape, transducer, and addition of liquid and gas can affect the final product in the sono-chemical reactor.^13,16,17^H_2_O → ultrasonic irradiation → ˙H + ˙OH˙OH + ˙OH → H_2_O_2_˙OH + ˙OH → H_2_ + O_2_
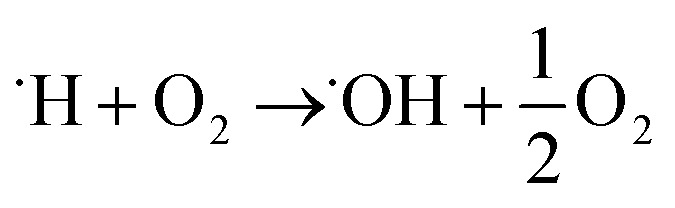

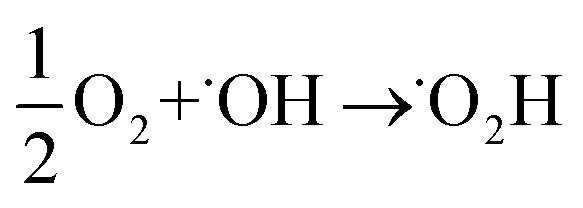
˙O_2_H + ˙H → H_2_O_2_˙O_2_H + ˙OH → H_2_O + O_2_C̲_6_H̲_8_O̲_7_ (lemon extract : citric acid) → thermal decomposition → carbon nanoplatesMg(NO_3_)_2_·6H_2_O → Mg^2+^ + 2NO_3_^−^Fe(NO_3_)_3_·9H_2_O → Fe^3+^ + 3NO_3_^−^Carbon nanoplates → ultrasonic irradiation → carbon nanoparticlesMg^2+^ + 2Fe^3+^ + carbon → ultrasonic irradiation → C@MgFe_2_O_4_ nanoparticlesC@MgFe_2_O_4_ nanoparticles → calcination → hollow MgFe_2_O_4_Hollow MgFe_2_O_4_ + carbon dot → ultrasonic irradiation → hollow MgFe_2_O_4_@carbon dot

By breakdown of bubbles that are created in solvent, micro-jets can break bonds and decrease accumulation.

Fig. 3b depicts a SEM image of carbon nanoparticles prepared under ultrasonic irradiation (150 W for 1 h), which confirms the presence of approximately mono-disperse nanostructures and shows their average size is around 60 nm.

Fig. 3c exhibits a SEM image of MgFe_2_O_4_ synthesized by the hydrothermal method. As we expected, MgFe_2_O_4_ has a hexagonal structure, and interestingly it is observed that micro-hexagons were synthesized. Our results prove precipitation is not effective for breaking of magnetic domains in this structure, so we applied higher temperature for removing of further materials, and outcomes show that by applying hydrothermal and sono-chemical methods, micro-structures are converted to nanostructures. Fig. 3d depicts a SEM image of MgFe_2_O_4_ after calcination (500 °C, 2 h) and sonication (150 W for 1 h). As particle size analysis verifies, the average size of the nanostructures is lower than 50 nm. With explosion in the bubbles, smaller compounds are obtained in hot small reactors. Our results confirm that by applying ultrasonic irradiation, tetragonal micro-structures are broken down and converted to semi-spherical nanoparticles with average diameter around 40 nm. For better dispersion, ultrasonic irradiation was performed after the solvo-thermal process. By increasing the temperature and accordingly the pressure in a closed reactor, carbon dots were appropriately synthesized, with an average size of about 30 nm.

**Fig. 4:**
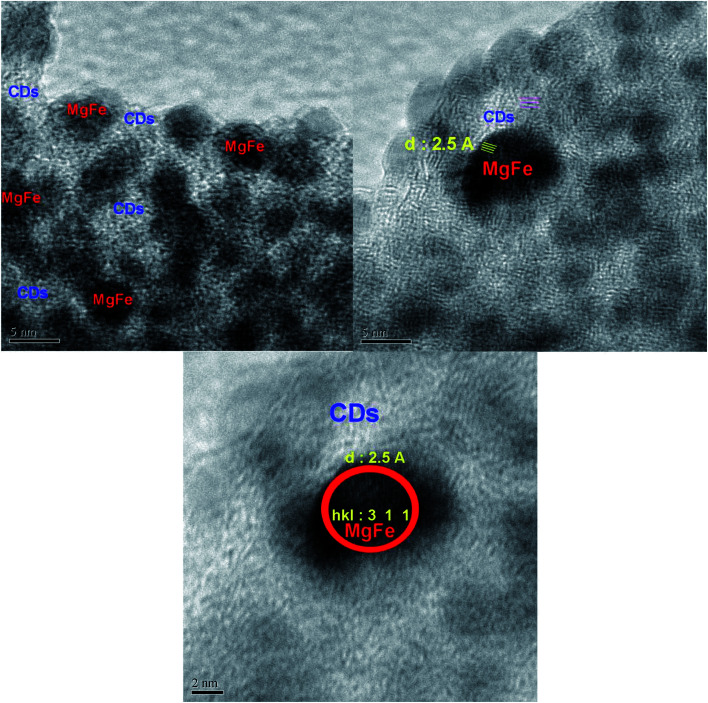
TEM images of MgFe_2_O_4_–carbon dot nanocomposite.

Fig. 4 illustrates transmission electron microscopy (TEM) images of magnesium ferrite–carbon dots. Interestingly, TEM images confirm magnesium ferrite is covered with carbon dots and a two-phase nanocomposite has been synthesized; high resolution TEM images show that the distance between crystal planes is about 2.5 Å, in agreement with XRD of pure standards. The images verify that the size of these porous nanostructures is less than 10 nm.

**Fig. 5:**
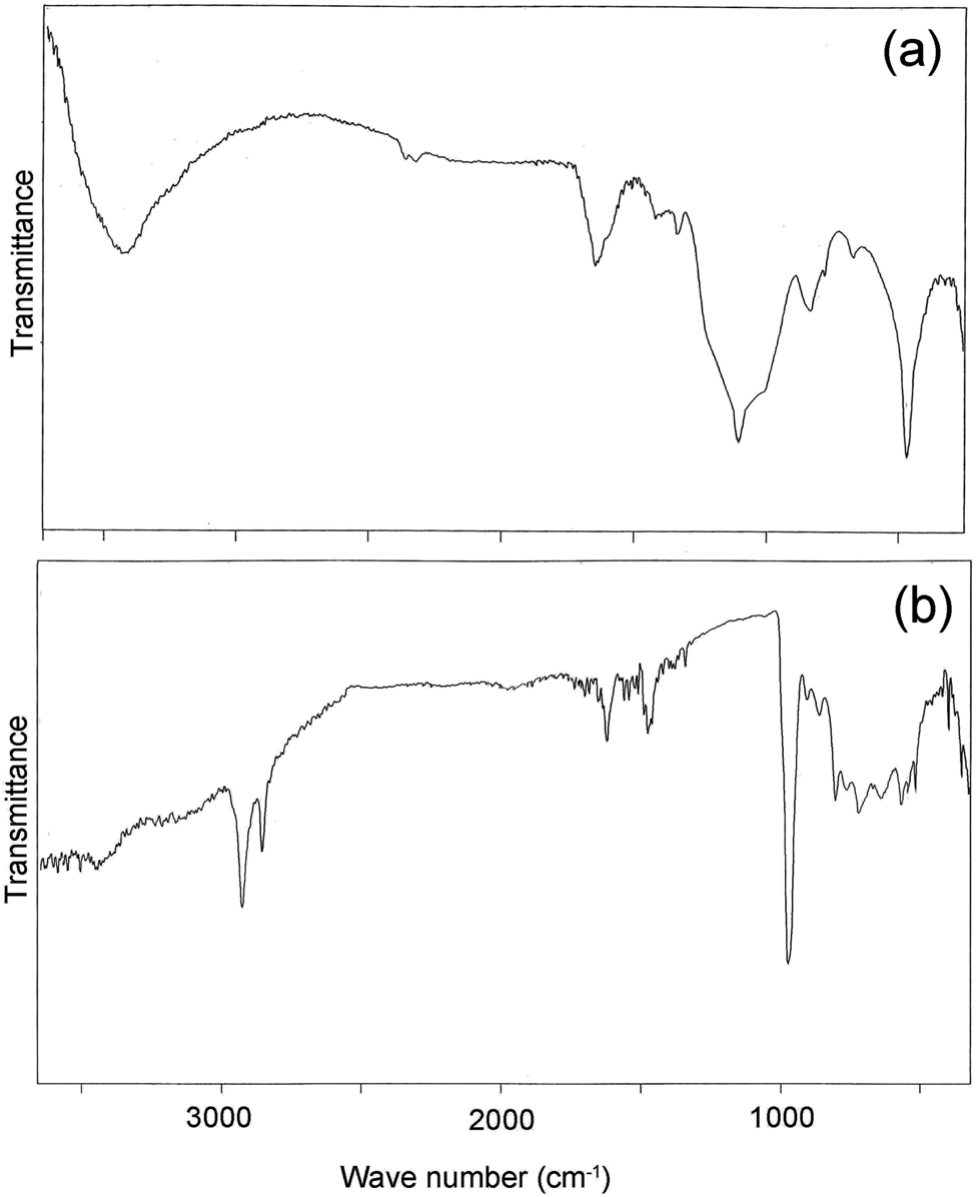
FT-IR curves of (a) MgFe_2_O_4_ nanoparticles, and (b) MgFe_2_O_4_–carbon nanocomposite.

Fig. 5a depicts Fourier transform infra-red spectra of the magnesium ferrite; the peak at 470 cm^−1^ is for Mg–O and Fe–O bonds, and the wide absorption at 3416 cm^−1^ is due to the O–H bond. The peak at 1103 cm^−1^ results from C–O bonds in citric acid. Fig. 5b illustrates the spectrum of the MgFe_2_O_4_–CDs nanocomposite; the presence of carboxyl and hydroxyl functional groups is demonstrated in this spectrum. The absorption at 972 cm^−1^ is attributed to C–O, and the bands at 2850 and 2920 cm^−1^ are for C–H bonds; the signal at 1620 cm^−1^ is due to C=O.

According to thermal gravimetric analysis (TGA), at temperatures of about 100–140 °C there is a small weight loss of around 5% due to hydroxyl groups and moisture on the hydrophilic structure, and there is also another obvious decrease of about 25% in the 350–420 °C range because of decomposition of the carbon dot coating; then from 400 to 800 °C the residual is constant owing to the thermal stability of the ferrite (metal-oxide).

**Fig. 6:**
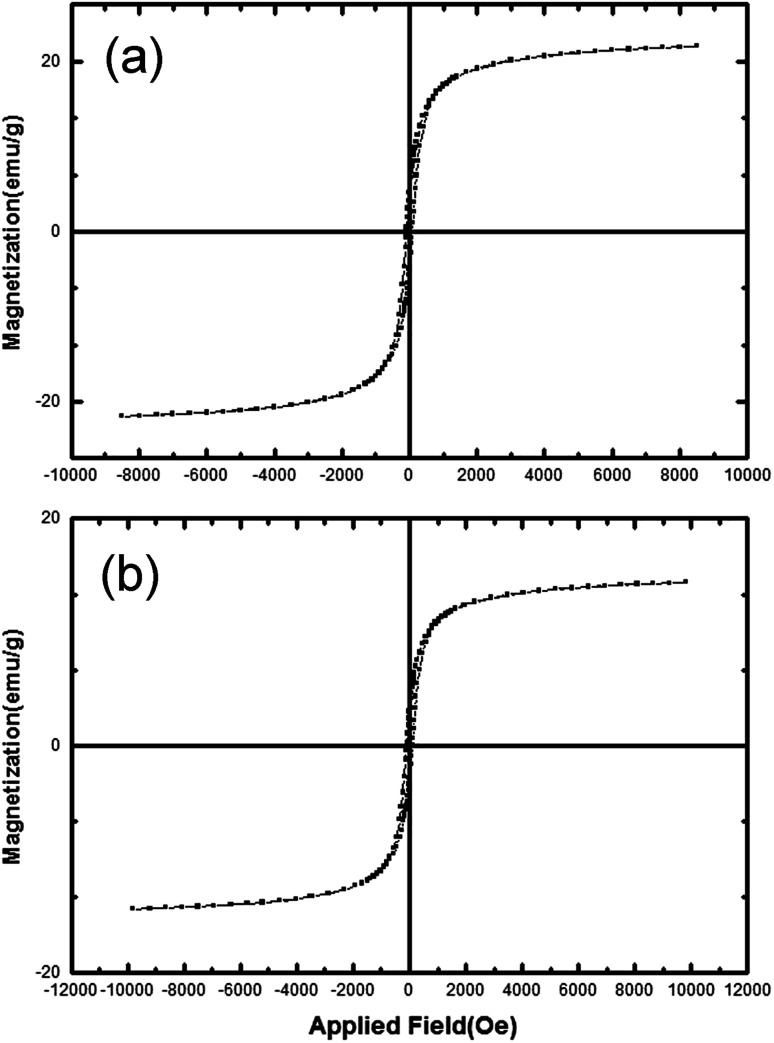
VSM curves of (a) MgFe_2_O_4_ nanoparticles, and (b) MgFe_2_O_4_–carbon dot nanocomposite.

A vibrating-sample magnetometer (VSM) was utilized to investigate magnetic properties; hysteresis loops for MgFe_2_O_4_ and MgFe_2_O_4_–carbon nano-compounds can be seen in Fig. 6a and b respectively. The nano-compounds display appropriate magnetic induction rendering them adequate for detection uses. The curves exhibit ferromagnetic behavior, with 22 emu/g saturation magnetization and 70 Oe coercivity for MgFe_2_O_4_, and 15 emu/g saturation magnetization and 100 Oe coercivity for the MgFe_2_O_4_–carbon nano-compound.

Covering magnesium ferrite with carbon dots increased the coercivity from 70 Oe to 100 Oe. Domains are stuck together in the process and so stronger magnetic field is needed to change their directions.

**Fig. 7:**
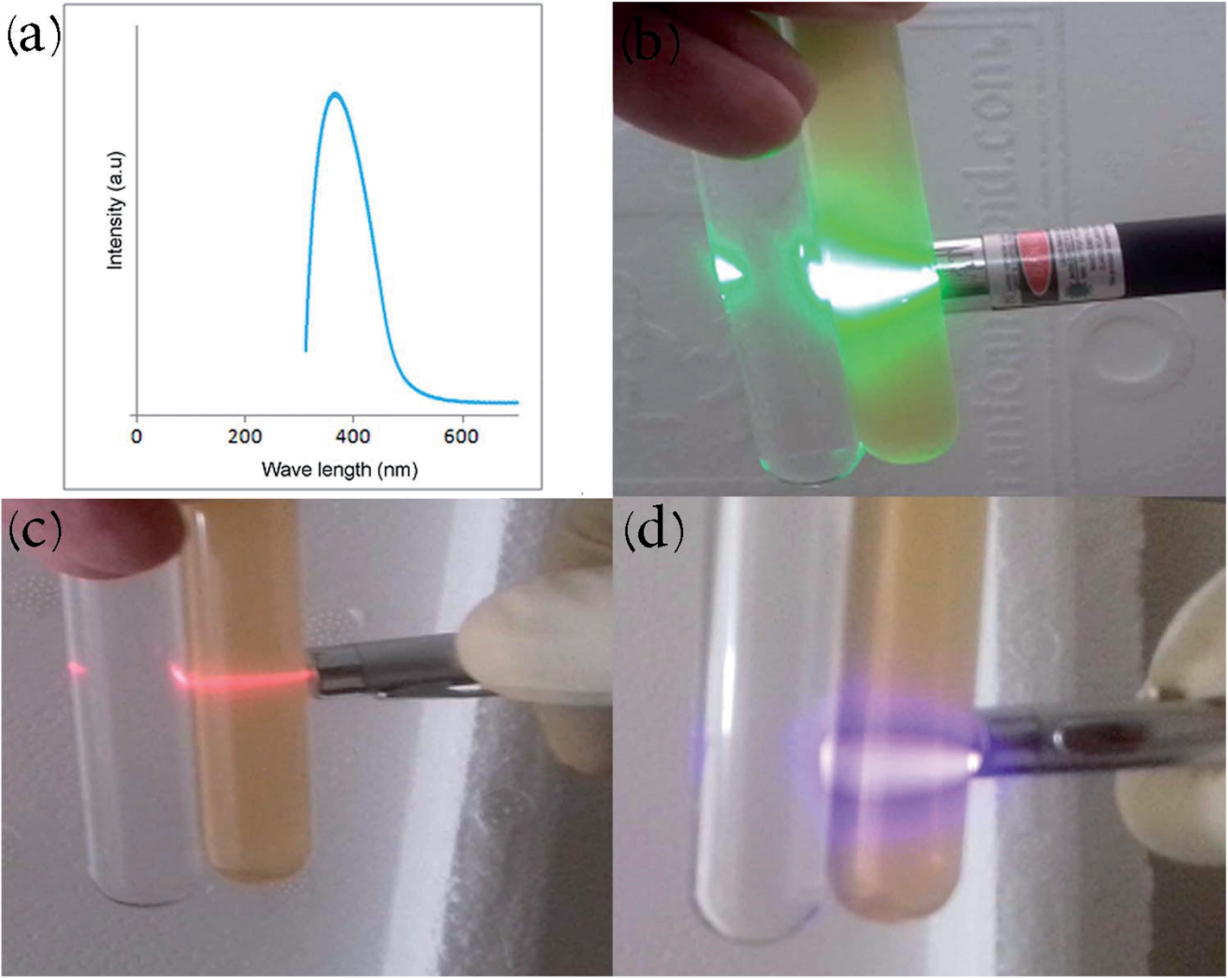
(a) UV-Vis spectrum of the carbon dot nanocomposite. Tyndall effect of carbon dots in water with (b) green, (c) red and (d) violet lasers.

The ultraviolet-visible (UV-Vis) absorption spectrum of the carbon dot nanocomposite prepared by the hydrothermal route at 200 °C is depicted in Fig. 7a. The compound displays an absorption peak at 380 nm (π–π* transition). CDs illustrate and achieve bandgap in comparison with conductive graphite due to quantum limitation, giving rise to UV absorption and PL peaks. Fig. 7b–d illustrate the Tyndall effect of the product in aqueous solution at different green, red and violet wavelengths. Beam scattering occurs when particles with approximately nano-dimensions are present in a solvent (colloid or nano-suspension). De-ionized water is shown beside the nanocomposite solutions for comparison. Electromagnetic radiation that has longer wavelength displays more transmission while shorter wavelengths exhibit more reflection.

**Fig. 8:**
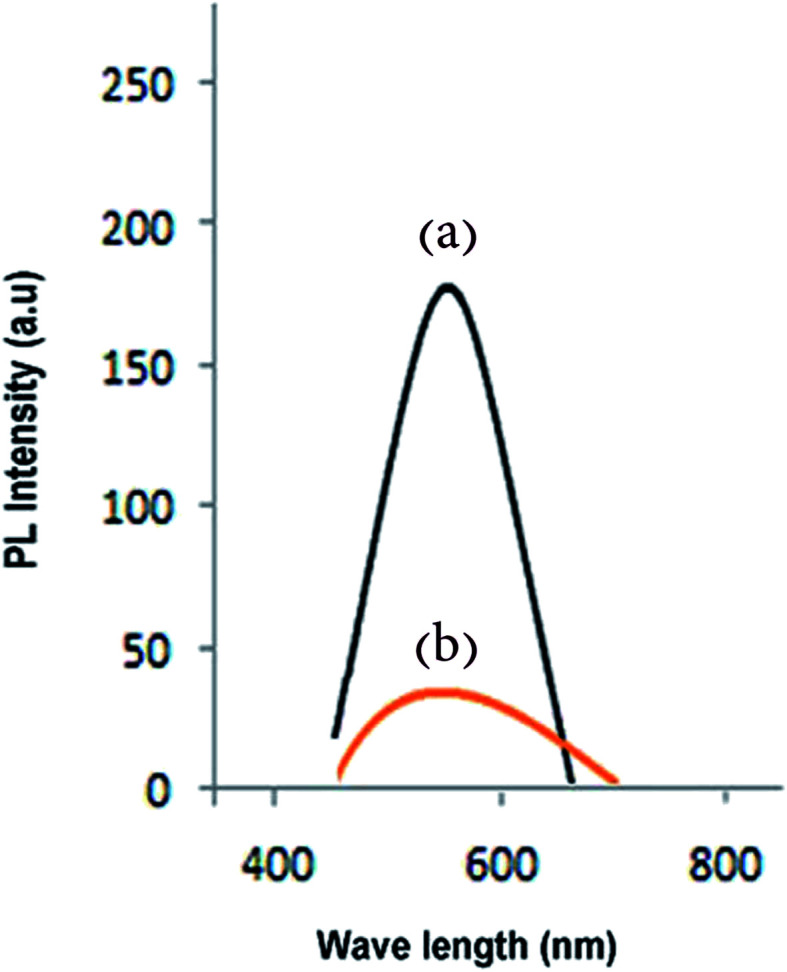
Quenching of PL intensity by addition of *Pseudomonas aeruginosa* bacteria.

**Fig. 9:**
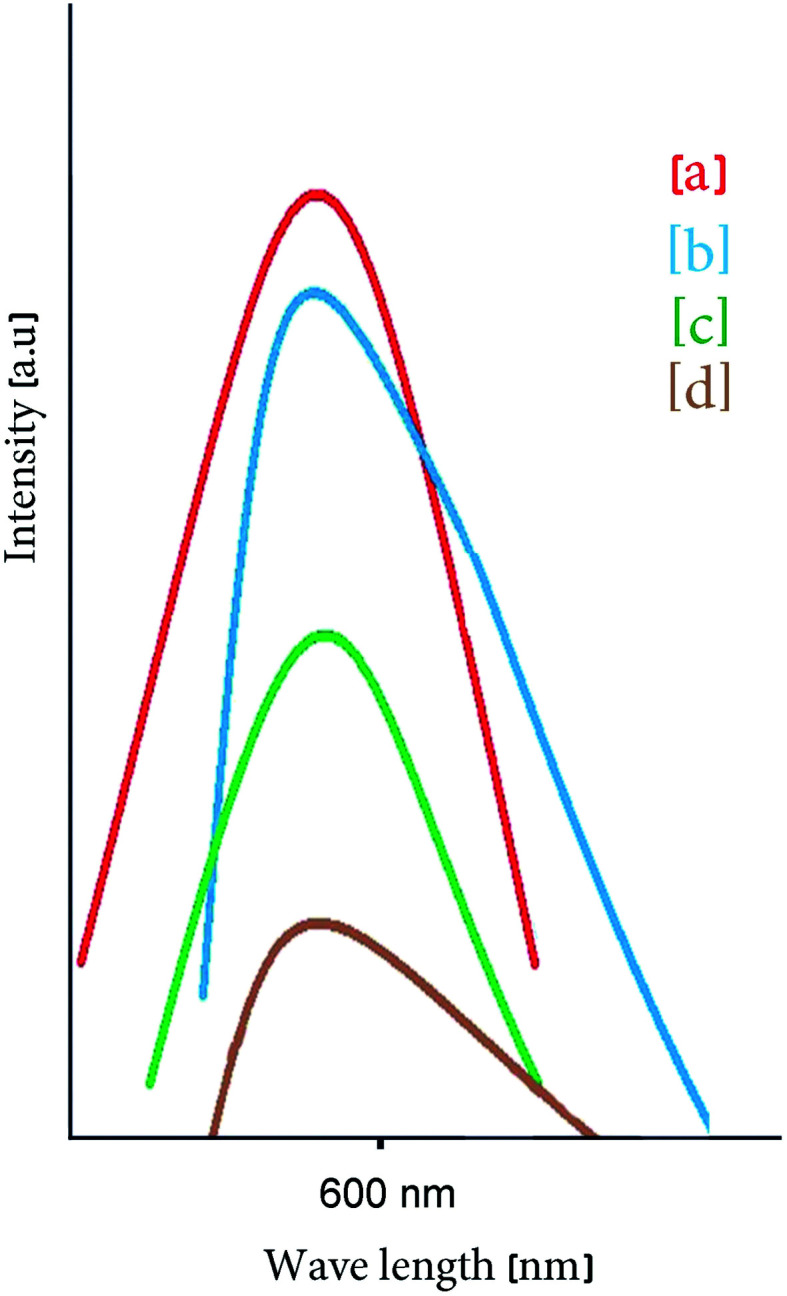
PL intensity of (a) pure carbon dots. Quenching by addition of toxic heavy-metal ions: (b) Ni(ii), (c) Cd(ii), (d) Hg(ii).

Luminescence spectra of carbon dots synthesized at 200 °C and reacted with *Pseudomonas aeruginosa* are displayed in Fig. 8; the excitation wavelength was about 450 nm and a PL peak at approximately 580 nm was obtained. The results verify a decrease in luminescence with increase in quantity of *Pseudomonas aeruginosa* (quantum yield around 18%).^1^ The surface electronic properties of the carbon nanostructure are changed by interaction with bacteria, extinguishing PL, which allows the concentration of the bacteria to be calculated.^1^ PL spectra of untreated carbon dots and carbon dots reacted with nickel(ii), cadmium(ii) and mercury(ii) are shown in Fig. 9a–d, respectively. Results confirm quenching of PL by increasing amounts with Ni(ii), Cd(ii) and Hg(ii) ions. The MgFe_2_O_4_–carbon carbon nanostructure is thus a suitable sensor for detecting toxic heavy metals. d-Orbitals of metal(ii) ions can accept excited electron from carbon dots. With the addition of ions, the fluorescence of the CDs is quenched because of the formation of complexes between ion(ii) and CDs (electron transfer process).^3^ PL spectra at various concentrations of CDs-labeled Cd(ii) were analyzed so that concentration of these hazardous ions could be measured.^1–4^

## 4. Conclusion

Carbon dots have attracted much attention due to their rare electron excitation–relaxation properties. Photoluminescence nanostructures were attached to a MgFe_2_O_4_ porous structure. Owing to pores in magnesium ferrite, carbon dots can diffuse to the inorganic Mg-ferrite core so we have simultaneously both magnetism and luminescence. The nanocomposite is feasible for sensing of harmful bacteria in waste water, and with the help of the magnetic core it can easily be collected after use. *Pseudomonas aeruginosa* and toxic metal ions can be detected by applying fluorescent carbon nanostructures.

## Conflicts of interest

There are no conflicts to declare.

## Supplementary Material
